# Positive and Negative Regulation of Ferroptosis and Its Role in Maintaining Metabolic and Redox Homeostasis

**DOI:** 10.1155/2021/9074206

**Published:** 2021-04-28

**Authors:** Ankita Sharma, Swaran Jeet Singh Flora

**Affiliations:** ^1^Department of Biotechnology, National Institute of Pharmaceutical Education and Research-Raebareli, Bijnor-Sisendi Road, Post Office Mati, Lucknow 226002, India; ^2^Department of Pharmacology & Toxicology, National Institute of Pharmaceutical Education and Research-Raebareli, Bijnor-Sisendi Road, Post Office Mati, Lucknow 226002, India

## Abstract

Ferroptosis is a recently recognized regulated form of cell death characterized by accumulation of lipid-based reactive oxygen species (ROS), particularly lipid hydroperoxides and loss of activity of the lipid repair enzyme glutathione peroxidase 4 (GPX4). This iron-dependent form of cell death is morphologically, biochemically, and also genetically discrete from other regulated cell death processes, which include autophagy, apoptosis, necrosis, and necroptosis. Ferroptosis is defined by three hallmarks, defined as the loss of lipid peroxide repair capacity by GPX4, the bioavailability of redox-active iron, and oxidation of polyunsaturated fatty acid- (PUFA-) containing phospholipids. Experimentally, it can be induced by many compounds (e.g., erastin, Ras-selective lethal small-molecule 3, and buthionine sulfoximine) and also can be pharmacologically inhibited by iron chelators (e.g., deferoxamine and deferoxamine mesylate) and lipid peroxidation inhibitors (e.g., ferrostatin and liproxstatin). The sensitivity of a cell towards ferroptotic cell death is tightly associated with the metabolism of amino acid, iron, and polyunsaturated fatty acid metabolism, and also with the biosynthesis of glutathione, phospholipids, NADPH, and coenzyme Q10. Ferroptosis sensitivity is also governed by many regulatory proteins, which also link ferroptosis to the function of key tumour suppressor pathways. In this review, we highlight the discovery of ferroptosis, the mechanism of ferroptosis regulation, and its association with other cellular metabolic processes.

## 1. Introduction

Cell death is an indispensable biological process that critically governs the development of multicellular organisms. It needs to be balanced with cell proliferation in order to maintain tissue homeostasis and also to prevent the onset of neoplastic diseases. Cell death is a catastrophic phenomenon which could be random and/or orchestrated. However, the type of cell death induced by different intracellular disorders or environmental stresses displays miscellaneous biochemical and morphological characteristics, which forms the basis of categorizing the distinguished types of cell death. The Nomenclature Committee on Cell Death (NCCD) has classified the process of cell death into two categories: accidental or regulated [1]. Cells experiencing extreme physical, chemical, or mechanical insults undergo the accidental cell death processes which could not be regulated by molecularly targeted interventions, such accidental cell death cannot be modulated. On the contrary, the regulated cell death processes are effectively controlled by pharmacological and biochemical interventions which could be modulated by molecular mechanisms. The cells which undergo the inflammatory path of cell death follow the necrotic cell death. The removal of damaged and unnecessary cells by an organized manner is termed as apoptosis. For several years, the only therapeutically tractable mechanism of regulated cell death was thought to be apoptosis [2]. However, the role of one of several regulated nonapoptotic cell death pathways, such as autophagy, necroptosis, pyroptosis, and ferroptosis, has also begun to emerge [1]. To reestablish the loss in homeostatic equilibrium, the exact identification of a specific cell death type is decisive. This review highlights the nonapoptotic cell death process of ferroptosis in greater details. Here, we discuss the hallmarks of ferroptosis, and its regulation by various biochemical and molecular players.

The term “ferroptosis” was coined by the lab of Dr. Brent R Stockwell in 2012 which describes a regulated cell death process which differs morphologically, biochemically, and genetically from the other forms of cell death processes: apoptosis, autophagy, and necrosis. Ferroptosis is a nonapoptotic, peroxidation-driven form of regulated cell death process identified more recently using a pharmacological approach which is characterized by the elevation of intracellular iron and oxidative stress generation in response to different stress stimuli [3]. The onset process of ferroptosis involves the iron-dependent enzymatic reaction-mediated generation of lipid soluble ROS. The detailed mechanisms of ferroptosis have extensively been studied in neurons, in glutamate-induced neurotoxicity, in organotypic hippocampal slice cultures, and in death signalling pathways induced by cerebral ischemia [4]. Iron chelators, inhibitors of lipid peroxidation, depletion of polyunsaturated fatty acids, and lipophilic antioxidants are found to work as effective suppressors of ferroptosis which have a direct correlation with the accumulation of lipid peroxidation markers.

## 2. Early History: Studies Linking Thiol Metabolism to Oxidative Cell Death

The history of identifying cell death with ferroptotic characteristics has been observed at various instances over the years before the detailed molecular understanding of this cell death process. Studies in the 1950s identified the essential role of extracellular cysteine and cystine for growth and proliferation of HeLa and other cells in culture [5]. The cell with cystine deficiency showed a distinct microscopic morphology which was similar to death caused by viral infection [6]. However, *α*-tocopherol, a lipophilic antioxidant, has shown to prevent the cell death induced in human fibroblasts cultured cells grown in cystine-free medium which led to reduction in cellular glutathione [7]. In the biochemical terms, ferroptosis is a process of lethal lipid peroxidation which involves oxidative addition of molecular oxygen (O_2_) to lipids. In 1955, the first few descriptions of such enzymatic reactions were shown by Mason et al. [8] and Hayaishi et al. [9] independently. In 1965, two separate groups suggested lipid peroxidation as a prime cause of cellular damage while studying drug toxicology in the liver of CCl_4_-treated rats [10, 11]. However, it was confirmed by 1980 that lipid peroxidation leads to the destruction of unsaturated lipid moieties of cell membranes, lipoproteins, and other structures leading to oxidative damage. The isolation of membrane-associated “phospholipid hydroperoxide glutathione peroxidase 4” (PHGPX or GPX4) which is the known second selenoperoxidase, after cytosolic glutathione peroxidase (GPX1) was a very significant discovery [12, 13]. In 1990s, GPX4, glutathione peroxidase, was shown to protect against peroxidative damage of biological membranes and phosphatidylcholine-containing liposomes which has now been identified as the enzymatic inhibition of ferroptosis [14–17]. The importance of GPX4 in according protection against cell lethality was established by many studies in transgenic and knockout mouse models of glutathione-dependent peroxidases [18–20, 21]. Seiler et al. in 2008 explained the role of 12/15-lipoxygenase (12/15-LOX), a polyunsaturated fatty acid metabolizing enzyme, in the execution of GPX4-knockout-mediated cell death which was the breakthrough in understanding of the unique properties of GPX4-downregulation-induced cell death [22].

This provided evidence of involvement of LOX in many fatal pathologic situations [23–26] and had laid the path to understand the mechanistic explanation of “oxidative stress-induced apoptosis,” independent of activation of Bcl-2 family proteins [27, 28]. This highlighted the need to identify a unique cell death mechanism which could link membrane oxidative stress, lipid peroxidation, arachidonic acid metabolism, and glutathione peroxidase activity (or lipophilic antioxidants; vitamin E).

**Figure 1:**
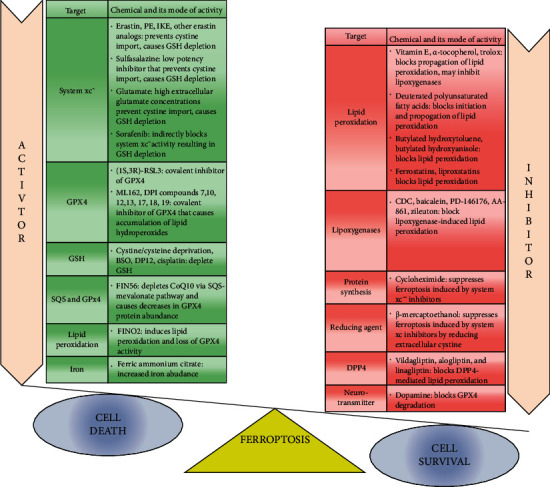
Modulation of ferroptosis by various chemical reagents. Ferroptosis is activated by four classes of ferroptosis inducers: (1) system xc− inhibitors, (2) GPX4 inhibitors, (3) FIN56, and (4) FINO2. Apart from these canonical ferroptosis inducers, many other reagents can also induce ferroptosis like buthionine sulfoximine (BSO) and ferric ammonium citrate. Ferroptosis is also inhibited by several pharmacological and genetic agents which inhibit lipid metabolism, lipid peroxidation, and iron metabolism. Vitamin E family (tocopherols and tocotrienols), small molecules, and flavonoids can inhibit LOX activity. Dopamine which is a neurotransmitter also leads to cell survival by blocking GPX4 degradation.

Reduced glutathione (*γ*-L-glutamyl-L-cysteinylglycine (GSH)) is an essential intracellular antioxidant synthesized from glutamate, cysteine, and glycine. This is a two-step process involving ATP-dependent cytosolic enzymes glutamate-cysteine ligase (GCL) and glutathione synthetase (GSS). Glutathione synthesis rate is largely dependent on cysteine availability. Cystine deficiency was shown to induce the serum-dependent cell death pathway, and the serum component critical for cell death was transferrin, an iron carrier. Early in the 1960s, iron was shown to contribute to lipid peroxidation-associated pathological changes in rats that could be prevented by vitamin E (tocopherols and tocotrienols), [29]. However, the addition of iron chelators, such as deferoxamine, and small molecules such as ferrostatin and liproxstatin prevents this cell death. These results established the concept that extracellular cystine and intracellular cysteine are required to maintain the biosynthesis of glutathione and to suppress a type of cell death in mammalian cells that is also preventable by treatment with iron chelators or lipophilic antioxidants. Also, the deprivation of cystine was found to be equivalent to system xc− inhibition. These findings provided the evidence for a unique form of regulated cell death mechanism induced by amino acid starvation in the presence of an exogenous iron source is ferroptosis (Figure 1).

## 3. Ferroptosis Hallmark Triad

Studies over the past decade have defined a core ferroptotic pathway that features the requirement for iron and the accumulation of reactive oxygen species (ROS). Both iron chelators and lipophilic antioxidants potently inhibit lipid ROS accumulation and cell death in response to ferroptosis inhibitors (erastin, RSL3, and other compounds). There are three core hallmarks to differentiate ferroptotic cell death from other forms of cell death mechanisms [30].

### 3.1. Oxidation of Phospholipids Containing Polyunsaturated Fatty Acid

Glycerophospholipids are the main components of the cell membranes of mammalian cells which are acylated with at least one polyunsaturated fatty acid (PUFA) chain. The phospholipid can be of thousands of types depending on chain lengths (C18 or higher), degrees of unsaturation (e.g., C18:3, C20:4, and C22:5), and also different head groups (e.g., phosphatidylethanolamine (PE), phosphatidylcholine, or phosphatidylinositol) in PUFA [31]. The deletion of LPCAT3 and ACSL4 genes which activates or incorporates the activated PUFAs into membrane PLs leads to the inhibition of ferroptosis [32–34]. Therefore, the inability of free PUFAs to promote ferroptosis is established which needs to be first activated and incorporated into membrane PLs to regulate ferroptosis [35]. Unlike saturated fatty acids and monounsaturated fatty acids, the bis-allylic hydrogen atoms present within PUFAs are highly susceptible to free radical (Gaschler & Stockwell 2017). PUFA oxidation is essential for ferroptosis in response to various stimuli like ROS and also enzyme-mediated oxidation [36, 37]. The oxidation of arachidonoyl (C20:4) or adrenoyl (C22:4) fatty acyl chains in the context of PE-PLs (i.e., PUFA-PE) is specifically linked to the execution of ferroptosis [38]. However, the molecular mechanism of cell death by oxidation of membrane PUFA-PEs and other species is not clear, but it is proposed that it could be by the formation of a structured lipid pore by membrane thinning and aberrant membrane curvature, as in the cases of necroptosis and pyroptosis [38, 39]. This signifies the availability of PUFA-PLs at a sufficient concentration as the first hallmark of ferroptosis. Lipid peroxidation preferentially oxidizes polyunsaturated fatty acids (PUFAs) which include linoleic, arachidonic, and docosahexaenoic acids. The PUFAs containing a (1Z, 4Z) pentadiene moiety are highly susceptible to peroxidation. The removal of hydrogen atom from methylene carbon is the first step of oxidation that bridges the two double bonds. As a result of resonance-stabilized pi system, the lipid isomerized into a more thermodynamically stable isomer (1Z, 3E) and conjugated diene which reacts with molecular oxygen to form a lipid peroxide [40].

### 3.2. Redox-Active Iron

Ferroptosis which is an iron-dependent form of cell death requires free iron or iron-containing lipoxygenase enzymes to oxidize membrane PUFAs, leading to the formation of lipid ROS. Free intracellular iron and the presence of membrane PL-PUFAs are critically required the initiation and execution of ferroptosis. Iron chelators (deferoxamine and ciclopirox) interfere with the generation of oxidized lipid species which thus inhibits ferroptosis [41]. Iron is imported into the cell as iron-transferrin complexes where the internalized transferrin receptor/transferrin-iron complexes localize to the lysosome [42]. Downregulation of cytosolic import of transferrin-iron complexes suppresses ferroptosis, because of decrease in cellular iron concentration [43, 44]. The acidic pH of the lysosome causes the release of iron from iron-transferrin complexes to the cytosol. In cytosol, ferritin nanocages are the major storehouses of iron, which gets released via NCOA4-dependent ferritinophagy [45]. CDGSH iron-sulfur domain 1 and the cysteine desulfurase NFS1 are the proteins responsible for utilization of iron for iron-sulfur cluster biogenesis. They tend to reduce the sensitivity of ferroptosis by preventing the accumulation of free, redox-active iron [46]. Therefore, the second hallmark of ferroptosis sensitivity is the presence of redox-active iron.

### 3.3. Loss of Lipid Peroxide Repair

The iron-catalysed PL-PUFA oxidation is a highly regulated process because of its potential toxic nature. Glutathione- (GSH-) dependent lipid hydroperoxidase specifically catalyses the reduction of lipid hydroperoxides to lipid alcohols in cell membranes [47]. GPX4 is an antioxidant enzyme preventing the accumulation of toxic lipid ROS during ferroptosis. GPX4 inactivation leads to the induction of ferroptosis in many cell types, whereas in mice, the deletion of this gene is found to cause lethality [48, 49]. Other small-molecule lipophilic antioxidants, like coenzyme Q10 (CoQ10) and vitamin E (*α*-tocopherol), also detoxify membrane lipid ROS. Unlike the endogenous antioxidants, several exogenous lipophilic antioxidants (e.g., ferrostatin-1 and liproxstatin-1) [3, 50] also inhibit ferroptosis by preventing the propagation of oxidative damage within the membrane [51]. Thus, the loss of the repair system for eliminating lipid hydroperoxides from PUFA-PLs comprises the third hallmark of ferroptosis.

## 4. Biochemical Regulation of Ferroptosis

**Figure 2:**
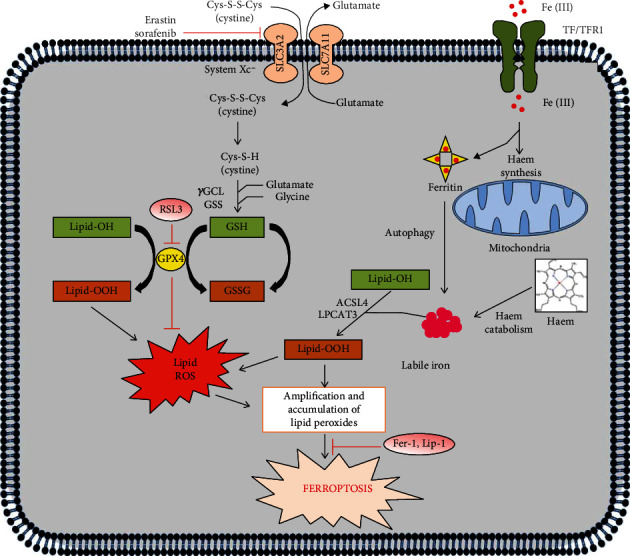
Regulation of ferroptosis by amino acid, lipid, and iron metabolism. The initiation and execution of ferroptosis are regulated by the metabolism of amino acid, lipid, and iron. The three hallmarks of ferroptosis are the presence of polyunsaturated fatty acid- (PUFA-) acylated phospholipids (PLs), redox-active iron, and loss in lipid peroxide repair. System xc− imports cystine, which gets reduced to cysteine to synthesize glutathione. GPX4 carries out the elimination of lipid peroxides by utilizing glutathione as a necessary cofactor. Transferrin and transferrin receptors are required for ferroptosis which import iron from the extracellular environment. Iron is essentially required for the accumulation of lipid peroxides. Stored Fe(III) in ferritin is retrieved by autophagy, and also, heme can be degraded by heme oxygenases (HOs) to recover Fe(II).

The initiation and execution of ferroptosis is a highly regulated process which lies at the juncture of lipid, amino acid, and iron metabolism (Figure 2).

### 4.1. Prerequisite of Amino Acid and Glutathione Metabolism in Ferroptosis Regulation

Since the biosynthesis of glutathione depends upon cysteine availability, therefore, metabolism of amino acid is intricately linked to ferroptosis regulation [52]. The cysteine is made available by the cytosolic import via the cystine/glutamate antiporter system xc−; however, some cells also utilize transsulfuration pathway to biosynthesize cysteine from methionine. This bypasses the requirement for cysteine making them resistant to ferroptosis induced by system xc− inhibitors. Glutamate and glutamine are also important regulatory roles in ferroptosis [53]. System xc− exchanges glutamate for cystine in a 1 : 1 ratio which thus impacts system xc− function. Higher concentrations of glutamate cause neurotoxicity since high extracellular concentrations of glutamate induce ferroptosis by inhibiting system xc− [3]. Thus, accumulation of extracellular glutamate serves to trigger the induction of ferroptosis in physiological contexts. Also, glutamine is present in human tissues and plasma at a higher concentration. The degradation of glutamine via glutaminolysis provides fuel for tricarboxylic acid (TCA) cycle and building blocks for other essential biosynthetic processes, like lipid biosynthesis. Also, *α*-ketoglutarate (*α*KG), a product of glutaminolysis, is required for ferroptosis [53]. Therefore, cellular conditions are when glutamine is absent, glutaminolysis is inhibited, or the depletion of cystine and/or blockage of cystine import does not lead to the accumulation of ROS, lipid peroxidation, and ferroptosis. However, not all the routes of glutaminolysis are needed for ferroptosis induction. GLS1 and GLS2 are the two glutaminases that catalyse the first step of glutaminolysis which is the conversion of glutamine into glutamate. Both these glutaminase are structurally and enzymatically similar, but still only GLS2 is required for ferroptosis which is also the transcriptional target of the tumour suppressor p53 [53, 54].

### 4.2. Intricate Association between Ferroptosis and Lipid Metabolism

Ferroptotic cell death is very intricately associated with the cellular lipid metabolism. As discussed earlier, PUFAs are highly susceptible to lipid peroxidation which is a prerequisite for the execution of ferroptosis. The availability and spatial localization of PUFAs determine the extent of cellular lipid peroxidation which has a direct relation with the execution of ferroptosis. Nonheme, iron-containing proteins, which includes lipoxygenases (LOXs), also act as enzymatic effectors mediating the ferroptotic peroxidation [22, 35, 37]. LOXs preferably catalyse the peroxidation of free PUFAs, rather than PUFA-containing phospholipids [55]. For this, PE phospholipids form a nonbilayer arrangement facilitating the proferroptotic oxidation of polyunsaturated fatty acid-containing PE phospholipids [56]. Genetic depletion of LOXs protects against erastin-induced ferroptosis, highlighting the contribution of LOXs in ferroptosis. Some lipoxygenases are also required for normal embryonic development in vertebrates. 12S-Lipoxgenase is indispensable for the development of numerous tissues in zebrafish which suggests that ferroptosis could also be involved in several developmental processes [57], since lipid peroxidation is the ultimate requirement for the execution of ferroptosis. Aldehydes and Michael acceptors are the reactive derivatives of lipid peroxides which react with nucleic acids and proteins [40]. The cell line selected for erastin resistance showed upregulation of aldo-keto reductase-encoding genes, AKR1C, which reduce lipid peroxidation reactive end-products to unreactive compounds [58].

### 4.3. Effects of Bioavailability of Iron on Ferroptosis

As the name suggests, iron is essential for ferroptotic cell death for the accumulation of lipid peroxides. The sensitivity of a cell towards ferroptosis depends on iron import, export, storage, and turnover. Transferrin and transferrin receptors, which are required for the iron import, are required for ferroptosis [4]. IREB2, the iron metabolism master regulator upon inhibition, decreases sensitivity to ferroptosis [3]. Since the iron metabolism is also impacted by autophagy, it also regulates ferroptosis in many ways [59]. Ferritinophagy is the selective autophagy of ferritin in which the ferritin is recognized by the specific cargo receptor NCOA4, which directs it to autophagosomes for lysosomal degradation. This lysosomal degradation of ferritin releases the free iron thus increasing the ferroptosis sensitivity [45]. Apart from ferritin, HSPB1 and CISD1 are other protein affecting the ferroptosis sensitivity. Thus, the regulation of ferroptosis by modulating iron metabolism and ferritinophagy is also of greater concern.

## 5. Molecular Regulation of Ferroptosis

**Figure 3:**
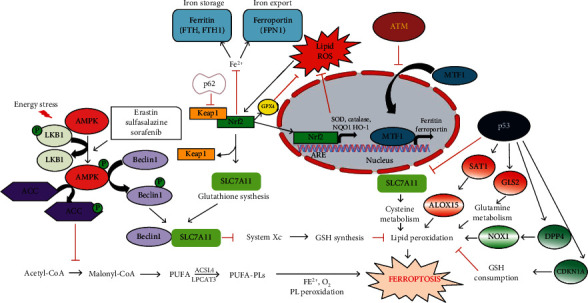
A schematic model describing the roles of various stress signalling pathways in the regulation of ferroptosis; p53 exhibits a dual regulatory roles in the control of ferroptosis. p53 increases ferroptosis either by inhibiting SLC7A11 expression or by activating SAT1 and GLS2 expression. p53-mediated inhibition of ferroptosis is mediated either by inhibiting DPP4 activity or inducing CDKN1A/p21 expression. ATM-mediated resistance to ferroptosis is induced by ATM inactivation. Upon ATM inactivation, the nuclear import of MTF1 protein is increased which transcriptionally activates the expression of ferroportin (FPN1) and ferritin (FTH1 and FTL) which leads to the presence of low-labile iron in the cell. Activation of the p62-Keap1- (Kelch-like ECH-associated protein 1-) Nrf2 (nuclear factor erythroid 2-related factor 2) pathway also regulates ferroptosis. Nrf2 plays the central regulatory role in the regulation of antioxidant molecules (HO1, NQO1) thus conferring protective against environmental or intracellular stresses. Nrf2 also controls the synthesis of GSH production which comprises an integral component of ferroptosis machinery. AMPK-mediated energy stress signalling pathway also has diverse roles in regulating ferroptosis. AMPK can either function by inhibiting major biosynthetic pathways (such as protein or fatty acid biosynthesis) or in association with Beclin-1 which is a key regulator of autophagy. BECN1 promotes ferroptosis by binding to SLC7A11 and thus blocking the system xc− activity.

Ferroptosis is characterized by the interplay of iron accumulation, excessive ROS levels, and lipid peroxidation where lipid hydroperoxide detoxification pathway activity is decreased. The biochemical mechanisms linking oxidative stress to mitochondrial dysfunction in ferroptosis have been elucidated and identified [60]. Therefore, genes and pathways that regulate iron homeostasis, endogenous lipophilic antioxidant pathways, PUFA metabolism, GPX4 expression, or GPX4 activity have emerged as imperative regulators of ferroptosis, since dysregulation of ferroptosis is also associated with many pathological conditions, because of its association with other biological processes such as immune responses [61], autophagy [62], and metabolism [63]. Therefore, few of the regulators of these biological processes which also affect the sensitivity of ferroptosis are explained in the following section (Figure 3).

### 5.1. Nrf2 Regulates All the Physiological Aspects of Ferroptosis

The nuclear factor erythroid 2-related factor 2 (Nrf2) is a key regulatory protein of the antioxidant response against the cellular oxidant imbalance [64]. Under normal physiological conditions, cellular Nrf2 is maintained in low amounts by its inhibitory protein, Kelch-like ECH-associated protein 1- (Keap1-) mediated proteasomal degradation. Under oxidative stress conditions, Nrf2 protein is stabilized and initiates a multistep pathway of activation that includes nuclear translocation, heterodimerization with its partner small v-maf avian musculoaponeurotic fibrosarcoma oncogene homolog (Maf) proteins such as MafG, recruitment of transcriptional coactivators, and subsequent binding to antioxidant response elements (ARE) of target antioxidant genes.

Nrf2 activation has been greatly linked to protection against cell death, but its role in the regulation of ferroptotic cell death and its relationship with iron signalling are the recently explored area. Nrf2 is found to transcriptionally regulate almost all genes implicated in ferroptosis which include NADPH regeneration which is critical for Gpx4 activity (phosphogluconate dehydrogenase, malic enzyme, and glucose 6-phosphate dehydrogenase) and genes for glutathione regulation (synthesis, cysteine supply via system xc−, glutathione peroxidase 4, and glutathione reductase) [65]. Nrf2 can also regulate the iron metabolism genes and attenuate ferroptosis through preventing free iron availability. The light chain and heavy chain of ferritin (FTL/FTH1), which is a key iron storage protein, and also ferroportin (SLC40A1), which regulates the efflux out of iron from the cell, are also regulated by Nrf2 [66, 67]. The Nrf2 is the vital inhibitor of ferroptosis due to its ability to inhibit cellular iron uptake, limiting ROS production and upregulating SLC7A11, which are indispensable for GSH synthesis [68].

Nrf2 also reciprocally regulates the metabolism of lipids mediated via ligand-mediated transcription factor peroxisome proliferator-activated receptor gamma (PPAR*γ*) [69, 70]. The relevance of PPAR*γ* in the regulation of ferroptosis is evident since it gets activated by oxidized lipids which are also relevant to the initiation of ferroptosis [71]. Hence, the regulation of ferroptosis by Nrf2 is indirect by modulating the lipids whose abundance contributes to the sensitivity to ferroptosis [32].

Nrf2 has recently been found to prevent the ferroptotic cell death by implying various direct mechanisms. Nrf2 regulates cellular iron and ROS metabolism via p62-Keap1-NRF2 pathway where it protects hepatocellular carcinoma cells against ferroptosis through upregulation of multiple genes (heme oxygenase-1 (HO1) and quinone oxidoreductase-1 (NQO1)). However, the knockdown of NQO1, HO1, and FTH1 followed by ferroptotic inducers increased the growth inhibition in HCC cells showing the negative modulatory activities of Nrf2 ferroptosis by its targeted genes [72]. Also, GPX4 which is a GSH-linked active ROS antagonist enzyme is one of the important targets of Nrf2 as mentioned above [73]. Moreover, inhibition of Nrf2 expression and activity *in vitro* and *in vivo* increased the anticancerous activity of erastin and sorafenib in HCC cells which are ferroptosis inhibitors [72, 74].

Ultimately, ROS is the indispensable molecule in the ferroptotic cell death. Previous studies have confirmed that miRNAs are intimately related to redox signalling and ROS production, which can therefore also regulate ferroptosis by regulating the expression of ROS. Nrf2 pathway is also readily activated by miR-7 and miR-200a which mediate the repression of Keap1 expression [75, 76]. On the contrary, the expression of Nrf2 is suppressed by miR-28 in a Keap1-independent way [77]. miR-101 and miR-455 target Cullin-3 (Cul3) to promote the nuclear accumulation of Nrf2 [78, 79]. Finally, miR-93 [80], miR-144 [81], miR-153, miR-142-5p, miR-27a [82], miR-193b, miR-29-b1 [83], miR-365-1, and miR-34a [84] also downregulate Nrf2 level through various mechanisms. This postulates the hypothesis that miRNA might modify ferroptosis by means of regulating the expression of Nrf2.

### 5.2. Beclin-1 Is a New Regulator of Ferroptosis

Beclin-1 (Vps30/Atg6 in yeast) is a recognized autophagy regulator that is involved in the generation of the PtdIns3K complex which is essentially involved in activating autophagy [85]. Beclin-1 also is a critical regulator of ferroptosis, which is independent of the formation of the PtdIns3K complex. Beclin-1 expression only affects system xc− inhibitor-induced ferroptosis. Knockdown of Beclin-1 by RNA interference (RNAi) blocks ferroptosis, whereas knocking of Beclin-1 by gene transfection promotes ferroptosis in cancer cells in response to system xc− inhibitors (e.g., erastin, sulfasalazine, and sorafenib). In contrast, suppression of a paralog of BECN1, Becn2 expression, does not affect erastin-, sorafenib-, or sulfasalazine-induced ferroptosis. This suggests that only Beclin-1 and not Beclin-2 is obligatory for system xc− inhibitor-induced ferroptosis [86].

Ferroptosis needs ATG5 (autophagy related 5) and NCOA4 (nuclear receptor coactivator 4). ATG5 is a part of an E3-like ligase which is critical for the lipidation of members of the GABARAP (GABA type-A receptor-associated protein families) and MAP1LC3 (microtubule-associated protein 1 light chain 3). However, NCOA4 is a cargo receptor which mediates FT/ferritin degradation via selective ferritinophagy. Suppression of *Atg5* by knockdown inhibits erastin-induced conversion of MAP1LC3B-I to MAP1LC3B-II. Also, suppression of NCOA4 blocks FT/ferritin degradation which results in suppression of ferroptosis. On the contrary, either knockdown or knocking of Beclin-1 does not affect the synthesis of lapidated MAP1LC3B and MAP1LC3B-positive puncta in ferroptosis. As a positive control in starvation-induced cells, knockdown of Beclin-1 stops conversion of MAP1LC3B-I to MAP1LC3B-II. Significantly, the formation of a BECN1-PtdIns3K complex was only observed in cancer cells in response to starvation, but not against ferroptotic stimulus. These findings point to the regulatory roles of Beclin-1 in ferroptosis as compared to starvation-induced autophagy [87].

The biochemical events which lead to ferroptosis majorly include lipid peroxidation and iron accumulation. However, the Beclin-1-mediated regulation of ferroptosis does not affect either intracellular iron accumulation or iron metabolism-associated expression. On the contrary, Beclin-1 promotes lipid peroxidation in the process of regulating ferroptosis. These findings suggest that BECN1 promotes ferroptosis also through regulation of lipid peroxidation [88].

### 5.3. Regulation of Ferroptosis by AMPK Is Context Dependent

AMP-activated protein kinase (AMPK), a critical sensor of cellular energy status, gets activated via AMP binding, upstream kinase phosphorylation, and other mechanisms. AMPK restores the energy balance and maintains cell survival under conditions of energy stress [89]. Since the unresolved energy stress has found to eventually induce apoptosis, the mechanism by which other nonapoptotic forms of regulated cell death are regulated is largely unknown. There are accumulating evidences which indicate an intimate relationship between metabolism and ferroptosis [41, 90].

Several *in vitro* studies have shown that the inactivation of AMPK greatly abolishes the protective effects of energy stress on ferroptosis. Also, the *in vivo* studies reveal the inhibition of ferroptosis-associated renal ischaemia-reperfusion injury upon AMPK inactivation. Cancer cells are shown to exhibit resistance to ferroptosis since they have a higher basal AMPK activation. However, these cells were found to be sensitized towards ferroptosis once there was physiological inhibition of AMPK activation, since AMPK also exhibits various regulatory roles in the lipid metabolism by mediating the phosphorylation of acetyl-CoA carboxylase and also polyunsaturated fatty acid biosynthesis. Importantly, loss of function of tumour suppressor liver kinase B1 (LKB1) sensitizes mouse embryonic fibroblasts (MEFs) and human non-small cell lung carcinoma cell lines to ferroptosis. This highlights the vital role of LKB1-AMPK-ACC1-FAS axis in regulating ferroptotic cell death [91]. Therefore, the functional and lipidomic analysis further links AMPK regulation to ferroptosis. Recently, a study has shown that mitochondria play an integral role in regulating ferroptosis [92]. AMPK activation is also found to regulate the mitochondrial homeostasis [93]. However, there are strikingly different functions between mitochondria and AMPK in ferroptosis, wherein mitochondria selectively promote erastin-induced or cystine-starvation-induced, but not RSL3-induced, ferroptosis [92], while energy stress-mediated AMPK activation inhibits ferroptosis through mitochondria-independent mechanisms [94]. The inhibitory effect of AMPK activation on ferroptosis does not involve the modulation of cystine uptake, iron metabolism autophagy, or mTORC1 signalling.

A recent study also reported a promoting role of AMPK in the regulation of ferroptosis mediated by Beclin-1. Beclin-1 is a key player of macroautophagic/autophagy, but at a few instances, it also plays a nonautophagic role in promoting ferroptosis by binding to SLC7A11. Notably, AMPK mediates the phosphorylation of Beclin-1 at Ser90/93/96. This is the prerequisite for the formation of Beclin1-SLC7A11 complex and subsequent lipid peroxidation in ferroptosis. Inhibition of AMPK by siRNA or compound C diminishes erastin-induced Beclin-1 phosphorylation at S93/96, which thus inhibits the formation of Beclin-1-SLC7A11 complex formation, and subsequent ferroptosis. Thus, it is evident that Beclin-1 contributes to the core molecular machinery and signalling pathways involved in ferroptosis [86]. It is possible that AMPK-mediated regulatory mechanisms of ferroptosis are context dependent and thus await for further investigation in future studies.

### 5.4. The Bifunctional Nature of Relationship between Tumour Suppressor Protein, p53, and Ferroptosis Network

Tumour suppressor protein p53 (TP53) plays a critical role in the cellular response to various stresses, including hypoxia, nutrition starvation, DNA damage, and oncogene activation [95]. p53 plays a dual role depending upon the stress conditions; it can either lead to cell survival or cell death including apoptosis and autophagy [96]. p53 has also been found to regulate ferroptosis at either transcriptional or posttranslational levels. p53 is found to exhibit both the properties; on one hand, it has prodeath function in ferroptosis, and on the other hand, it also shows prosurvival properties in ferroptosis [97]. p53-induced ferroptotic cell death is mediated by the inhibition of SLC7A11 expression, promotion of SAT1 expression, and promotion of GLS2 expression [98]. The transrepression of SLC7A11 expression by p53 activation is found to promote ferroptosis in fibroblasts and other cancer cells (human osteosarcoma U2OS and human breast cancer MCF7). For instance, p533KR, an acetylation-defective mutant with 3 lysine residues (in positions 117, 161, and 162) getting replaced by arginine residues, is found to be highly effective in repressing SLC711A without affecting the other regulatory effects of p53 like the regulation of cell cycle or apoptosis [99]. On the contrary, p534KR98, which is an acetylation-defective mutant in which an addition lysine in position 98 has been replaced, is incapable of reducing the expression of SLC711A [100]. In human cancers, the high levels of the oncogenic E3 ubiquitin protein ligase MDM2 degrade the wild-type p53 which is regarded as a promising therapeutic strategy for the treatment of cancer [101]. As expected, the level of p53 is increased in MDM2-/- cells. The embryonic lethality observed in MDM2-/- mouse embryos is ferroptotic cell death, because this effect was significantly reversed by the administration of ferroptosis inhibitors like ferrostatin-1 [99].

SAT1 (spermidine/Spermine N1-acetyltransferase 1) is an important regulator in polyamine metabolism. Oxidative stress, inflammatory stimuli, and heat shock are found to upsurge the activity of SAT1 which associates the abnormal SAT1 expression or impaired polyamine metabolism to various pathological conditions, including cancer [102]. Several *in vitro* studies have proved that SAT1 is also a transcriptional target of p53. No other cell death inhibitors (Z-VAD-FMK, necrostatin-1, and 3-methyladenine) but only ferrostatin-1 is found to inhibit ROS-induced cell death in SAT1 Tet-on cells. Although the expression and/or activity of SLC7A11 and GPX4 are not altered by SAT-1, the induction of SAT1 is in corelation with ALOX15 (arachidonate 15-lipoxygenase). This is mechanistically confirmed by the pharmacological inhibition of ALOX15 by PD146176 which represses the SAT-1-mediated ferroptosis. SAT1 depletion also has an inhibitory effect on p53- and p533^KR^-induced ferroptosis [103].

GSL2 (glutaminase 2), a mitochondrial glutaminases, is also an important target that regulates ferroptosis. As glutamine metabolism is essentially involved in ferroptosis, the first step of glutamine catabolism involves the conversion to glutamate, which is catalysed by mitochondrial glutaminases [104]. GSL2 has been recently identified as a transcriptional target of p53 and is responsible for p53-mediated oxygen consumption, mitochondrial respiration, and ATP generation in cancer cells. Also, several *in vitro* studies have also highlighted the GLS2 mediated increase in cellular antioxidant function through increased GSH production [105]. According to these instances, GLS2 should act as a negative regulator of ferroptosis, but contrary to this, the knockdown of GLS2 in fibroblasts inhibits (but not promotes) serum-dependent ferroptosis through control of glutaminolysis [53]. Therefore, the involvement of GLS2 in ferroptosis needs to be explored further to identify the specific requirement of GLS2 in erastin or RSL3-induced ferroptosis or the p53-induced ferroptosis.

As stated above, p53 has bifunctional roles in the regulation of ferroptosis. The death-inducing potentials of p53 have been enumerated above; the prosurvival function of p53 will be discussed further. The regulation of localization and activity of DPP4 (dipeptidyl peptidase-4) forms the major regulatory mechanism exhibiting the prosurvival function of p53. Inhibition of p53 by either by knockdown or by pharmacologic interventions sensitizes the cancer cells to type I ferroptosis inducer (erastin and SAS), but not to type II ferroptosis inducer (RSL3 and FIN56). However, DPP4 inhibitors (linagliptin, vildagliptin, and alogliptin) completely block erastin-induced cell death in p53-deficient cells which could not be achieved by other protease inhibitors (doxycycline, ritonavir, atazanavir, VX-222, semagacestat, Z-FA-FMK, odanacatib, Z-VAD-FMK, and DAPT) [106]. The tumour suppressor CDKN1A/p21 (cyclin-dependent kinase inhibitor 1A), a key mediator of p53, also has prosurvival functions in response to oxidative stress by inhibiting apoptosis [107]. In the conditions of cystine deprivation in cancer cells, p53-mediated CDKN1A expression delays the onset of ferroptosis. Also, the inhibition of MDM2 by nutlin-3 increases the expression of p53 which blocked system xc− inhibitor-induced ferroptosis [108]. Remarkably, it is seen that the CDK4/6 inhibitors are not able to block ferroptosis representing that CDKN1A-mediated cell cycle arrest is not enough to inhibit ferroptosis. The mechanism of action of CDKN1A in ferroptosis still needs to be explored in greater depths to assess its role in the development and treatment of cancer.

### 5.5. Signalling Downstream of Ataxia-Telangiectasia-Mutated Kinase in Ferroptosis Regulation

Ataxia-telangiectasia-mutated kinase (ATM) is a kinase crucial for the DNA damage responses [109]. p53 is one of its downstream targets which plays a very decisive role in regulating ferroptosis which prompts its role in the ferroptosis [110]. Inhibition of ATM either by genetic knockdown or by pharmacologically suppressing ferroptotic cell death also decreases intracellular labile iron via increasing iron export (increased FPN1) and sequestration (increased FTH1 and FTL). This synchronized change in the iron regulators during ATM inhibition results in the depletion of labile iron pool which thus prevents the iron-dependent ferroptosis. These coordinated changes rely greatly on transcriptional activity and nuclear translocation of metal-regulatory transcription factor 1 (MTF1) upon ATM inhibition. Under the conditions of ATM inhibition, the nuclear translocation of MTF1 is increased which leads to inhibition of ferroptosis by the alterations in the expression of ferritin (FTH1) and ferroportin (FPN1). In conditions where activity of MTF-1 is depleted, the cells show a greater sensitivity to ferroptosis signifying the prevalence of ATM-MTF1-ferritin/FPN1 regulatory axis regulating labile iron levels [111].

## 6. Conclusion and Future Direction

Ferroptosis as the name suggests is a unique regulated form of the cell death pathway associated with iron overload, with many of its physiological roles yet to be defined. The molecular mechanism of ferroptosis is far more complex than previously thought and is entirely different from other regulated cell death processes including apoptosis, necroptosis, and autophagy. Extensive research which deals with understanding the fine regulatory mechanisms of ferroptosis is growing with a very fast pace. However, many studies have also shown the contradictory relationship of ferroptosis with other types of cell death types. Several regulatory proteins have been identified recently which regulate ferroptosis by either direct or indirect mechanisms with many targeting iron metabolism and lipid peroxidation. Many of these ferroptosis regulators are also associated in other types of RCD. Therefore, the imperative objective in the study of ferroptosis is the identification of executors or immediate downstream signalling pathways which might distinguish ferroptosis from other types of RCD. This will elucidate the further molecular controls of this process and also explain its involvement in various diseases, and its physiological and evolutionary functions.

## Abbreviations

12/15-LOX: 12/15-Lipoxygenase
15-LOX: 15-Lipoxygenase
AA: Arachidonic acid
ACC: Acetyl-CoA carboxylase
ACSL4: Acyl-CoA synthetase long chain family member 4
AKR1C: Aldo-keto reductases
AMPK: AMP-activated protein kinase
ARE: Antioxidant response elements
ATG: Autophagy related gene
ATM: Ataxia-telangiectasia mutated kinase
CDKN1A/p21: Cyclin-dependent kinase inhibitor 1A
CoQ10: Coenzyme Q10
Cul3: Cullin-3
DPP4: Dipeptidyl peptidase-4
FPN1: Ferroportin
FTH1: Ferritin
GABARAP: GABA type-A receptor-associated protein
GCL: Glutamate-cysteine ligase
GLS: Glutaminases
GPX: Glutathione peroxidase
GSH: *γ*-L-Glutamyl-L-cysteinylglycine
GSS: Glutathione synthetase
HO1: Heme oxygenase-1
Keap1: Kelch-like ECH-associated protein 1
LPCAT3: Lysophosphatidylcholine acyltransferase 3
Maf: Musculoaponeurotic fibrosarcoma
MAP1LC3: Microtubule-associated protein 1 light chain 3
MTF1: Metal-regulatory transcription factor 1
NCCD: Nomenclature Committee on Cell Death
NCOA4: Nuclear receptor coactivator 4
NQO1: Quinone oxidoreductase-1
Nrf2: Nuclear factor erythroid 2-related factor 2
PE: Phosphatidylethanolamine
PHGPX: Phospholipid hydroperoxide glutathione peroxidase
PL: Phospholipid
PPAR*γ*: Peroxisome proliferator-activated receptor gamma
PUFA: Polyunsaturated fatty acid
PUFA-PL: Polyunsaturated fatty acid-containing phospholipids
PUFAs: Polyunsaturated fatty acids
ROS: Reactive oxygen species
RSL3: Ras-selective lethal small-molecule 3
SAT1: Spermidine/Spermine N1-acetyltransferase 1
SLC7A11: Solute carrier family 7 member 11
TP53: Tumour suppressor protein p53.
